# New Screening Protocol for Effective Green Solvents Selection of Benzamide, Salicylamide and Ethenzamide

**DOI:** 10.3390/molecules27103323

**Published:** 2022-05-22

**Authors:** Maciej Przybyłek, Anna Miernicka, Mateusz Nowak, Piotr Cysewski

**Affiliations:** Department of Physical Chemistry, Pharmacy Faculty, Collegium Medicum of Bydgoszcz, Nicolaus Copernicus University in Toruń, Kurpińskiego 5, 85-950 Bydgoszcz, Poland; 282892@stud.umk.pl (A.M.); 294597@stud.umk.pl (M.N.)

**Keywords:** solubility, green solvents, benzamide, salicylamide, ethenzamide, binary solvents, COSMO-RS, intermolecular interactions, affinity

## Abstract

New protocol for screening efficient and environmentally friendly solvents was proposed and experimentally verified. The guidance for solvent selection comes from computed solubility via COSMO-RS approach. Furthermore, solute-solvent affinities computed using advanced quantum chemistry level were used as a rationale for observed solvents ranking. The screening protocol pointed out that 4-formylomorpholine (4FM) is an attractive solubilizer compared to commonly used aprotic solvents such as DMSO and DMF. This was tested experimentally by measuring the solubility of the title compounds in aqueous binary mixtures in the temperature range between 298.15 K and 313.15 K. Additional measurements were also performed for aqueous binary mixtures of DMSO and DMF. It has been found that the solubility of studied aromatic amides is very high and quite similar in all three aprotic solvents. For most aqueous binary mixtures, a significant decrease in solubility with a decrease in the organic fraction is observed, indicating that all systems can be regarded as efficient solvent-anti-solvent pairs. In the case of salicylamide dissolved in aqueous-4FM binary mixtures, a strong synergistic effect has been found leading to the highest solubility for 0.6 mole fraction of 4-FM.

## 1. Introduction

Amides have been widely used in pharmacy as can be inferred from 649 records found in the DrugBank database [1,2,3]. Some examples are bacteria and antiviral agents (oseltamivir, cephalexin, ampicillin), anesthetics (lidocaine, bupivacaine, prilocaine), analgesics and nonsteroidal anti-inflammatory drugs (acetaminophen, salicylamide and ethenzamide). Some benzamide analogs for example salicylamide and ethenzamide are known for several decades and are used as over-the-counter medications in cases of headaches, migraine, cold and fever [4,5,6]. Technically speaking these active pharmaceutical ingredients (API) are orto-substituted analogs of Benzamide and share several common features. All have moderate hydrophilicity (logP < 3) and relatively low solubility in water, alcohols and other dissolution media. However, their weak dissolution abilities can be overcome through cocrystallization [7,8]. Importantly, the studies on the solid-state complexes showed that amides can interact very efficiently with aliphatic and aromatic carboxylic acids [7,8,9,10,11,12]. The basic properties of amides are associated with the resonance effect contributing to the electron density increase on the carbonyl oxygen atom [13,14]. Nevertheless, due to this effect, the N–H bond is more polarized, which implies proton-donating abilities enhancement. Hence, amides can form relatively strong intermolecular hydrogen bonds with proton-accepting compounds such as DMSO [15,16,17,18], DMF [18], acetone, tetrahydrofuran (THF) [19], acetonitrile [18,20] and 1,4-dioxane [18].

Solubility is one of the basic properties characterizing pharmaceuticals. This can be explained by the relationships between solubility and bioavailability, which have been extensively studied [21,22,23,24]. Nevertheless, there are various technical issues closely associated with drug manufacturing, which cannot be underestimated. Indeed, solvents are used in every important step of drug development including synthesis, crystallization, extraction, and final form development. According to the Pharmaceutical Roundtable of American Chemical Society’s Green Chemistry Institute (ACSGCI), organic solvents comprise an average of 54% of chemicals and materials used in the technological processes of the pharmaceutical industry, and hence the recycling, waste reduction, azeotropy (energy costs reduction during evaporation) and solvent safety are issues of particular importance in the context of sustainable pharmaceuticals’ production design [25]. The high solubilizing ability, which enables the dissolution of a wide range of organic compounds, is also an important feature that distinguishes green solvents, especially when considering extraction processes. [26,27]. For this reason, it is crucial to develop strategies for selecting solvents that meet efficiency, safety, environmental hazard, and human health protection requirements. In recent years there has been growing interest in exploring solubility and partitioning properties of various compounds. A special attention should be paid for pharmaceuticals and nutraceuticals in green solvents such as ionic liquids [28,29,30,31,32], bio-based solvents including natural deep eutectic solvents (NADES) [33,34,35,36,37,38,39] and some environmentally friendly synthetic organic solvents, such as ethanol or DMSO and their organic-organic and organic-aqueous mixtures [40,41,42,43]. The application of multicomponent solvents containing water are interesting because it is possible to replace the environmentally hazardous pure organic solvent with less harmful aqueous mixtures. Noteworthy, although water is often considered an anti-solvent compared to organic solvents, in some cases, it can enhance the solubility, as a result of a significant co-solvation effect. Such synergism was frequently observed in many aqueous binary mixtures as for example for sulfamethizole in 1,4-dioxane [40,44] or acetonitrile binary mixtures [40], sulfanilamide in ethanol, acetonitrile and 1,4-dioxane [45,46], nicotinamide in DMSO and acetonitrile [18], phenacetin in 1,4-dioxane [47,48], amoxicillin in DMF-water [49] and theophylline in aqueous DMF, DMSO, 1,4-dioxane, 1-propanol and 1-butanol [33,50,51]. 

It is worth emphasizing that due to a large number of neat and binary aqueous solvents potentially interesting from the pharmaceutical practice, it is difficult to find the most optimal ones using time-consuming experimental methods. Hence, theoretical screening enabling the rational selection of the most promising candidates seems indispensable. Unfortunately, despite great efforts [18,33,40,45,52,53,54,55,56,57,58,59,60], there is no universal and reliable way of predicting solubility in binary aqueous solutions from the molecular structure. Methods developed for proper back-computations of measured solubility data such as Jouyban-Acree, van’t Hoff-Yaws, Wilson, Apelblat, Buchowski-Ksiazczak and Non-Random Two Liquid (NRTL) are interesting from the thermodynamic interpretation viewpoint [45,61,62,63,64], however rather of limited applicability as screening tools. Hence, the first principle approaches seem to be attractive, even if prediction accuracies are only semi-quantitative or qualitative [55,65,66] Among many of them the COSMO-RS methodology [67], is a very powerful tool applied for predicting various physicochemical properties using exclusively information of the chemical formula. In addition to many molecular affinity-related properties such as activity coefficients [68,69], equilibrium constants [70,71,72], cocrystals and solvates screening [10,11,67,73,74,75,76,77], phase diagrams [78,79,80,81,82,83,84,85,86,87], solubility in neat and multicomponent solvents [18,33,40,45,55,61,74,88,89,90,91,92,93,94,95], solubility parameters estimation [96,97,98,99] and partition coefficients [92,100,101,102] were computed with varying success. Nevertheless, this method is relatively efficient and allows determining the thermodynamic characteristics of mixtures based on the optimized 3D molecular structure. The use of molecular modeling methods facilitates directing experimental efforts to a limited population of systems, thereby allowing for saving time and chemicals. The latter aspect is of particular importance in the context of green and sustainable strategies. 

The aim of this study is threefold. Firstly, the pool of experimental solubility data is extended by providing new results obtained for titled compounds in two proton accepting organic solvents (DMSO and DMF) and their aqueous mixtures. The choice of DMSO and DMF was mainly guided by previous research showing their high efficiency in the case of other amides and sulfonamides [18,40,45,47]. Secondly, the accuracies of solubility computed using the COSMO-RS methodology were carefully checked against available experimental data of benzamide, salicylamide, and ethenzamide. Finally, the obtained model was used for finding efficient and environmentally friendly aqueous binary mixtures and new solubility measurements were performed for gaining information on solubility and external validation of the proposed screening protocol.

## 2. Results and Discussion

Solubility of the three selected aromatic amides was already the subject of serious interest as compiled in Table 1. The provided list encompasses pure water and 20 neat organic solvents. Additionally, ethenzamide solubility was determined by Tong et al. [103] in thee binary mixtures of acetonitrile with either methanol, ethanol, or isopropanol. This study also provides the solubility in neat components. The other two aromatic amides were not studied in any mixed solvents. 

The equilibrium in the solid-liquid saturated systems is governed not only by the properties of solvents but also by the solid state including polymorphic or pseudopolymorphic transformations as well as solvates formation. Hence, the precise description of the solids is an important aspect of solubility determination. For this reason, the sediments collected after the shake-flask experiments for water and studied organic solvents were examined using differential scanning calorimetry (DSC) and Fourier transform infrared spectroscopy–attenuated total reflectance (FTIR-ATR) techniques. The detailed characteristics of solid residues were provided in supplementary materials (see Figures S5–S10). In the case of solvate formation or polymorphic transformation, the absorption band shifts on the IR spectra are often related to new hydrogen bonding patterns. In the case of DSC thermograms, new phases can be detected by observing additional signals due to the heat transfer associated with polymorphic transition or pseudo-polymorph transformations. It happened that in all studied cases no physical change of the solids is observed since both IR spectra and DSC thermograms recorded for the sediments do not differ significantly from those recorded for pure reagent. Based on the available literature data and the measurements performed in this study, it can be concluded that benzamide, salicylamide, and ethenzamide do not show a tendency to undergo phase changes in aqueous and organic solutions. This conclusion is in good accord with already reported observation. According to Ouyang et al. (2019) benzamide solid form is not altered in contact with such solvents as water, methanol, ethanol, 1-propanol, isopropanol, 1-butanol, isobutanol, methyl acetate, ethyl acetate, butyl acetate, acetonitrile and acetone [9]. In the case of salicylamide, the only polymorphic transformation observed so far was with the use of high pressure [110]. According to studies on the solubility and crystal form of salicylamide in water, methanol, ethyl acetate, acetonitrile, acetone, and acetic acid [104,105], no solid phase polymorphic or pseudo-polymorphic transitions were observed. As reported by Wang et al. (2021) [108] no polymorphic transitions of ethenzamide were observed in all studied sediments coming from saturated solutions in isopropanol, 1-butanol, 2-butanol, 1-pentanol, methyl acetate, ethyl formate, propyl acetate, butyl acetate, DMF, acetone, 1,4-dioxane and 2-butanone). The same observation was also reported for ethenzamide in methanol, ethanol, 1-propanol, isobutanol, ethyl acetate, and acetonitrile [103,106].

### 2.1. Benzamide

**Figure 1:**
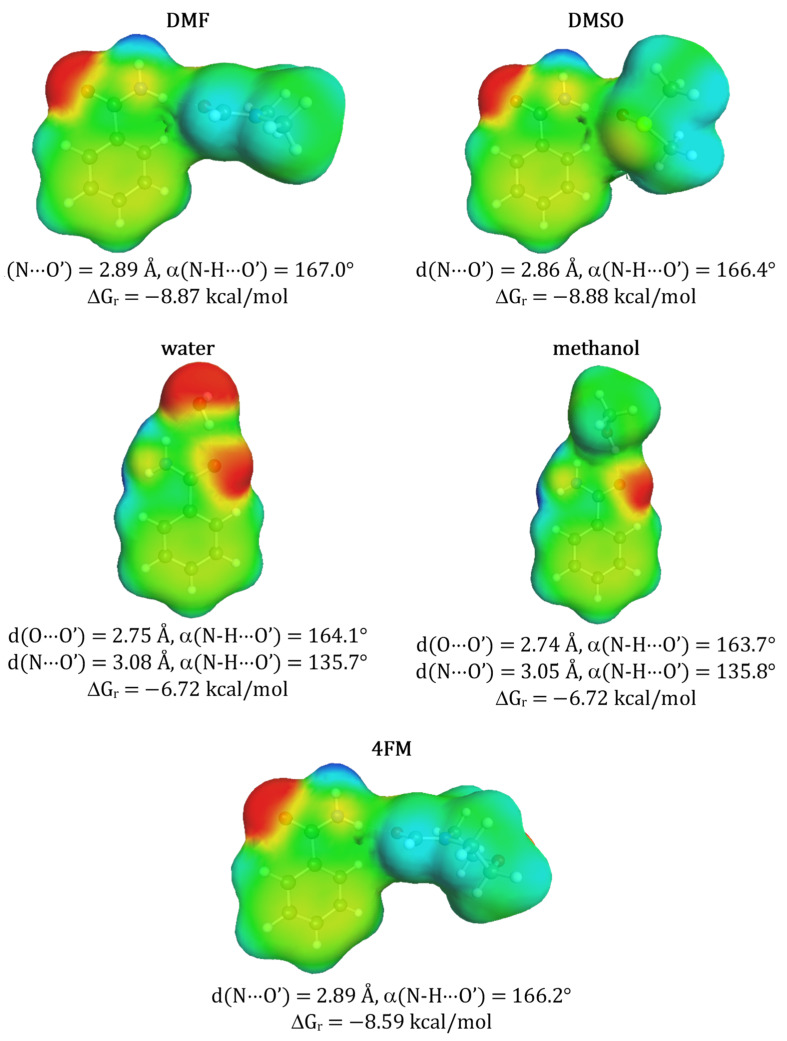
Structural and thermodynamic characteristics of selected solute-solvent contacts of benzamide. The affinity measure (ΔG_r_) represents concentration-independent value associated with activity constant of the corresponding reaction of pair formation at room temperature. Prime denotes atoms belonging to solvent molecules.

As enumerated in Table 1 the solubility of benzamide was experimentally determined in 13 solvents in a broad range of temperatures. So far the highest solubility at ambient conditions was reported for neat methanol (x_B_ = 0.092) [9] and the lowest for water (x_B_ = 0.002 [9]) and at elevated temperatures solubility typically slightly increases. It is rather quite expected that this modest solubility can be optimized using other not studied so far solvents. In many cases, the qualitative criteria of alternative solvents selection can be proposed based on chemical intuition. Indeed, the amide group can be considered as a proton-donating center active with proton-acceptor solvents. There are several popular solvents of this type commonly used for solubility measurements such as DMSO or DMF. Due to the absence of corresponding experimental data for benzamide, these two solvents were chosen as the first choice for further experimental investigations and to expand the pool of available solubility data. These two solvents are characterized by high efficiency in dissolving many solids, including active pharmaceutical ingredients [18,40,45,47,49,111,112]. Their solubilizing potential can be interpreted in terms of solute-solvent affinity and in the particular case of benzamide the expected structures and corresponding energetics, determined theoretically, are documented in Figure 1. According to the results of quantum chemistry computations quite high affinity of benzamide is expected for either of these two solvents. In addition, ketones and esters such as acetone or methyl acetate are expected to exhibit similar properties. However, benzamide affinity toward these aprotic solvents is significantly lower which is reflected by reduced solubility. On the other hand, interactions of benzamide with amphiprotic water and proton donating methanol are different. These two solvents form two-center hydrogen bonding with both parts of the amide group as exemplified in Figure 1. The value of ΔG_r_ for methanol is comparable to DMSO and DMF. Although, there is not any general straight relationship noticed between affinities and solubility it is interesting to notice that for a subclass of aprotic solvents there is a modest linear dependency with R^2^ ≈ 0.9. This fortunate circumstance supports DMF and DMSO selection as potentially good solvents for benzamide. Unfortunately, the former is definitely problematic from the environmental impact perspective. In general, DMF is considered a toxic compound causing hepatic disorders [113]. Although DMSO is much less hazardous, and is by some studies considered a green solvent [114,115,116], there are reports on its side effects indicating the need for its replacement with greener alternatives [117,118]. It has been already documented [40] that morpholine analogs belonging to the same class of solvents as DMSO can be regarded as a real alternative to DMSO not only as an effective solubilizer but also from the perspective of environmental impact and costs of measurements. Indeed, previous studies documented that 4FM is a very promising solvent that meets the criteria of environmental friendliness and was found to be a greener alternative for DMF [40,119,120,121,122,123,124]. However, there is very little research on its solubilizing properties. A very few examples that can be found in the literature is solubility studies on methane, hydrogen sulfide, carbon dioxide [125], ethane [126], propane [127], and caffeine [55]. For this reason, it is worth exploring the solubility of benzamide and also other studied aromatic amides in 4FM and their mixtures with water. Additionally, the results of affinity computations support the selection of 4FM as a potential solubilizer of benzamide. The corresponding values provided in Figure 1 suggest both the same nature of solute-solvent interactions and high affinity as DMSO.

**Figure 2:**
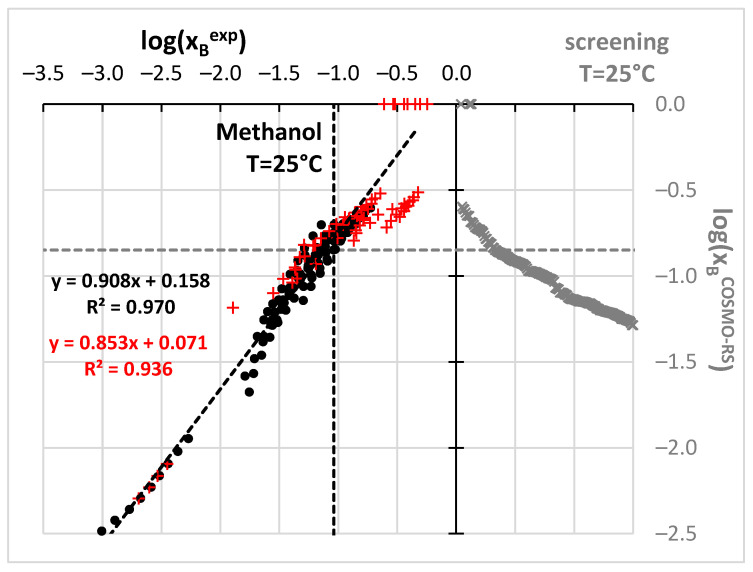
Results of benzamide solubility measurements and computations. All experimental points marked as black circles were taken from the literature while new measurements are presented as red plusses. Computed solubility values are presented in gray color. Dashed lines stand for best solubility values estimated so far at room temperature.

**Figure 3:**
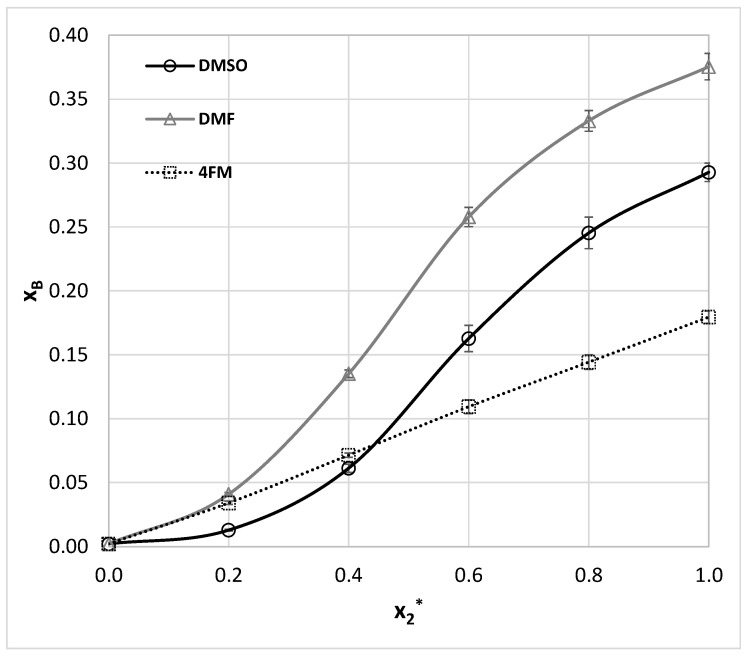
The results of benzamide solubility measurements (molar fraction, x_B_) at room temperature in aqueous DMSO, DMF, and 4FM mixtures (x_2_* stands for the organic component mole fraction in the binary solvent). The error bars denote standard deviation values (*n* = 3).

However, before the actual selection of suggested aprotic solvents for benzamide solubility measurements additional and more qualitative screening has been performed based on the values of solubility computed using the COSMO-RS approach. The possibility of carrying out ab initio predictions directly from the molecular structure is very appealing [90]. Unfortunately, COSMO-RS formalism despite relying on chemical intuition, often fails in determining solubility [93,128,129,130,131] and other physicochemical properties [132,133,134,135]. Probably in most cases, the major explanation is the insufficient precision of available default parameterization [128,136], which on the other hand, in many cases provides quite an accurate match with measured values [56,90,91,137]. Fortunately, non-substituted benzamide is a relatively simple molecular system, which seems to be well-described by COSMO-RS. Indeed, the correlation between computed and experimental solubility (Figure 2) was found to be very satisfactory. Points marked with black ink represent the trend obtained for the set of already measured and published data. The data obtained for m-xylene were not included in the analysis since these old measurements correspond to much higher temperatures compared to other sets. The highly linear relationships between experimental and computed values enable extended screening of neat solvents not studied so far with the hope of finding better ones than already identified. Hopefully, this reduces the number of experiments to be performed. There are many potential green solvents which might be considered in such screening, since there are many various classification systems [123,138,139,140,141,142,143,144]. Here, the pool of solvents taken into account was adopted from the EPA (Environmental Protection Agency) [145] list provided by the solvent substitution Paris III software tool [146,147,148,149,150] suitable for evaluating the environmental impact of solvents. This protocol relies on the assessment of the overall environmental index (EI) including estimated parameters modeling the expected potential of human toxicity by ingestion (HTPIng), human toxicity by inhalation (HTPInh), terrestrial toxicity (TTP), aquatic toxicity (ATP), global warming (GWP), ozone depletion (ODP), photochemical oxidation (PCOP) and acid rain (AR). The initial set of 5423 solvents included in the Paris III program was shortened by imposing some practical restrictions. First of all, it is expected to find real and practical alternatives to commonly used solvents. Hence, those solvents which are not commercially available were excluded. This shortened the list to 3399 solvents. The second criterion was the environmental impact quantified based on indices proposed by EPA. The span of EI values ranged from 0.02 for water up to more than a million for chloranyl(methoxy)methane and chloranyl(chloromethyloxy)methane. The latter compounds are considered very toxic. A very tight criterion was imposed on solvent “greenness” by including only such solvents, for which EI < 2. This resulted in 758 solvents, for which solubilities of benzamide were computed in 298.15 K. Noteworthy, such a tight criterion excluded the two initially selected solvents (DMSO and DMF). The computed solubility values assorted according to decreasing values of log(x_B_^est^) were represented by gray crosses in the right part of Figure 2. All points placed above the gray dotted line indicate solvents assumed to be better than methanol and are supposed to be more efficient dissolving media for benzamide. It happened that the predicted benzamide solubility in DMSO is infinite, which is obviously incorrect. There are also other solvents for which the same outcome was computed as for example for 2-methoxyethanamine CAS = [109-85-3], diethylene glycol bis(3-aminopropyl) ether (CAS= [4246-51-9]) or tetren CAS = [112-57-2]. Despite the lack of actual solubility values, this prediction can be interpreted as an indication of the very high solubility of benzamide in these solvents. In addition, the solubility predicted in DMF is very high (log(x_B_^est^) = −0.60). What is the most interesting the solubility of benzamide in 4FM seems to be also very high, log(x_B_^est^) = −0.61. Hence, qualitative guidance based on chemical intuition, the nature of solute-solvent interactions and qualitative screening led to the same conclusion that 4FM might be a real alternative to other solvents. Taking advantage of this suggestion experimental work was carried out for Benzamide solubility determination in 4FM. Additionally, for comparison purposes, the solubility of benzamide was measured also in DMSO and DMF. Apart from the neat solvents, a series of aqueous binary mixtures were considered in four different ratios of water and organic solvent. In Figure 3 the benzamide molar fraction solubility values corresponding to 298.15 K were presented. All measured solubility data are summarized in supplementary materials (Table S1). It is worth concluding that a systematic increase in solubility is observed with the increase of concentration of all solvents irrespectively of temperature. Moreover, the lack of synergistic effect and typical water antisolvent behavior shows that neat organic solvents are the best solubilizes. Their solubilizing power can be ordered as follows: DMF > DMSO > 4FM. 

To evaluate the green potential of experimentally studied water-organic solvents, the EI analysis was performed (Table 2). Each binary mixture was analyzed twice. Values corresponding to the default weighting of all contributions to EI were augmented with ones excluding of PCOP index in the analysis. This is justified by the fact that DMSO is commonly accepted as a green solvent but has a very high PCOP contribution. It is quite reasonable to ignore this parameter since PCOP is associated with the photochemical degradation in the atmosphere, which seems to be less important considering quite low volatility of DMSO and the absence of leaks in the technological equipment. Such simplification was already postulated [40,55]. Since, water is the most environmentally friendly solvent (EI = 0.02), with an increase in its proportion, the “green” potential of used solvents increases. Unfortunately, in most cases, solubility follows the opposite trend. If all the parameters are taken into account 4FM is the highest-ranked neat organic solvent. However, if the PCOP index is omitted then the DMSO-water mixture (x_2_ = 0.2) is supposed to be the most environmentally friendly. 

### 2.2. Salicylamide

**Figure 4:**
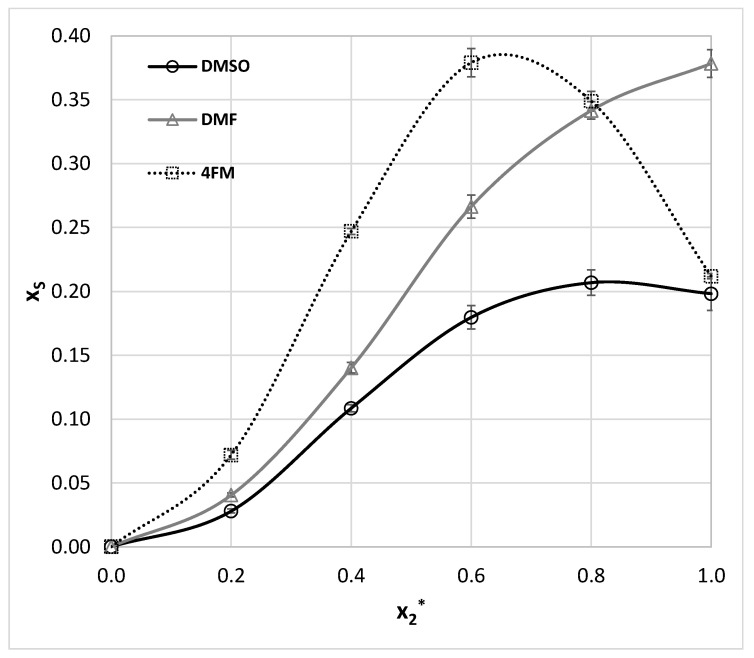
The results of salicylamide solubility measurements (molar fraction, x_S_) at room temperature in aqueous DMSO, DMF, and 4FM mixtures (x_2_* stands for the organic component mole fraction in the binary solvent). The error bars denote standard deviation values (*n* = 3).

Salicylamide was studied in a much-extended set of solvents compared to benzamide. Unfortunately for many systems only room temperature solubility was measured [107] and no data for aqueous-organic solvents mixtures are available. Hence, the pool of salicylamide solubility data was extended by measurements in DMSO, DMF, and mixtures with water in the range of temperatures between 298.15 and 313.15 K. The obtained results are collected in supporting materials (see Table S2) and in Figure 4 for room temperature. According to available literature data, the best solvent for salicylamide found so far is THF, for which x_S_ = 0.176 [9]. As it was documented in Figure 4 both DMF and DMSO act as much more efficient solubilizes than THF and even significant dilution with water still leads to higher solubility potential.

**Figure 5:**
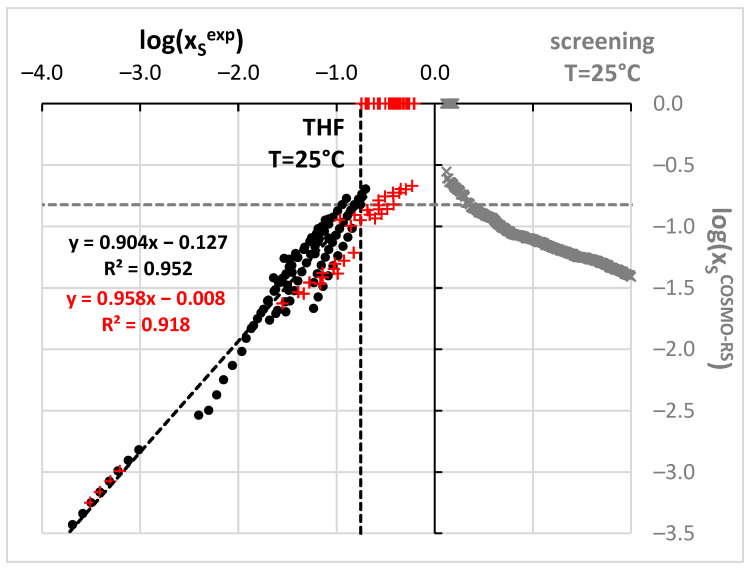
Results of salicylamide solubility measurements and computations. Notation is the same as in Figure 2.

Although this finding is interesting on its own, it is worth following the same approach as applied to benzamide for screening of alternative solvents. In Figure 5 there is presented a relationship between computed and measured solubility values. The obtained correlation is quite satisfactory both for neat and binary solvents mixtures. However, despite the fact that the trend seems to be linear, there are many systems for which solubility is predicted with substantial inaccuracy. For example, solubility in acetic acid is seriously underestimated with a mole fraction error of magnitude −60%. In addition, salicylamide dissolution behavior in many solvents is misinterpreted as infinitely soluble. Among these problematic systems, one can find solvents with a high concentration of DMSO and DMF. On the contrary solubilities in alcohols are generally predicted by COSMO-RS with satisfactory accuracy. Nonetheless, the computed values are still informative and it is really interesting to point out that several solvents were identified as better compared to THF. It is not surprising that due to high structural similarities between salicylamide and benzamide 4FM was found as a potentially effective solvent. It is interesting to notice that a significant co-solvation effect occurs for the 4FM mole fraction of 0.6. This mixture is an effective solvent for salicylamide as neat DMF and the corresponding molar fraction solubility reached 0.379. Moreover, it is expected to be far more environmentally friendly (EI = 0.46) than pure DMF (EI = 2.16) (Table 2).

**Figure 6:**
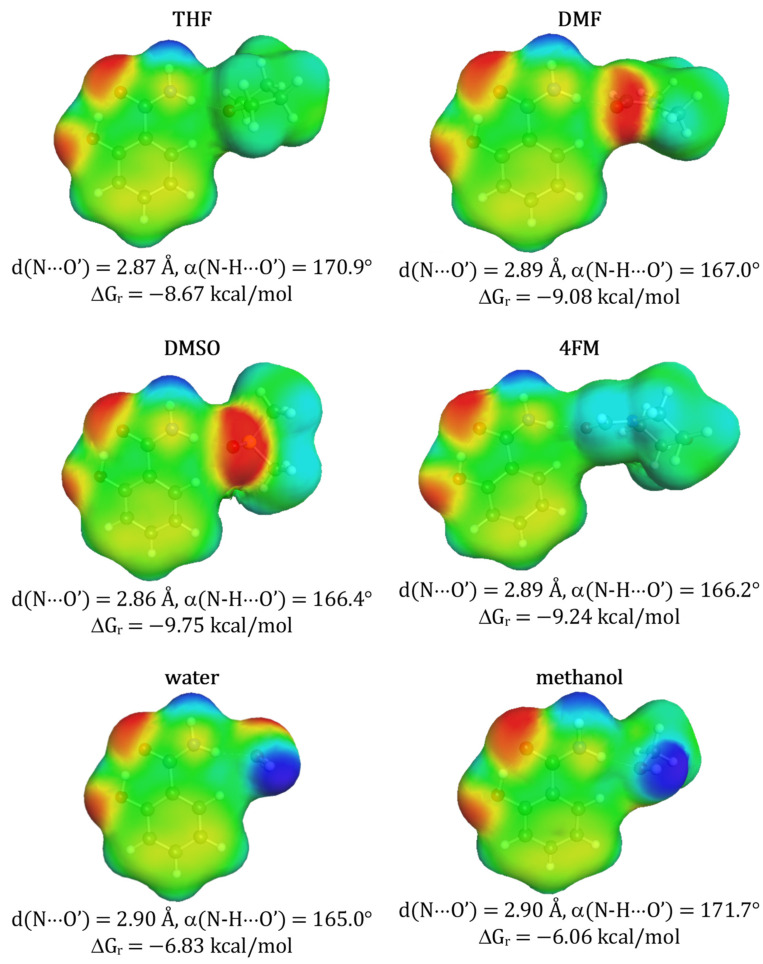
Structural and thermodynamic characteristics of selected solute-solvent contacts of salicylamide. Notation is the same as in Figure 1.

Such high solubility of salicylamide in all three studied aprotic solvents can be explained by the solute-solvent affinities documented in Figure 6. The pairs formed between salicylamide and solvent molecule are stabilized by a strong hydrogen bond formed between the amide group acting as a donor and the electronegative center of the solvent molecule acting as acceptor. The affinity of salicylamide to DMSO was found to be the highest which nicely corresponds with observed solubility. Other aprotic and proton accepting solvents have similar affinities. The values of Gibbs free energy of hetero association are significantly smaller for water and methanol reflecting the significantly lower solubility of these solvents.

### 2.3. Ethenzamide

**Figure 7:**
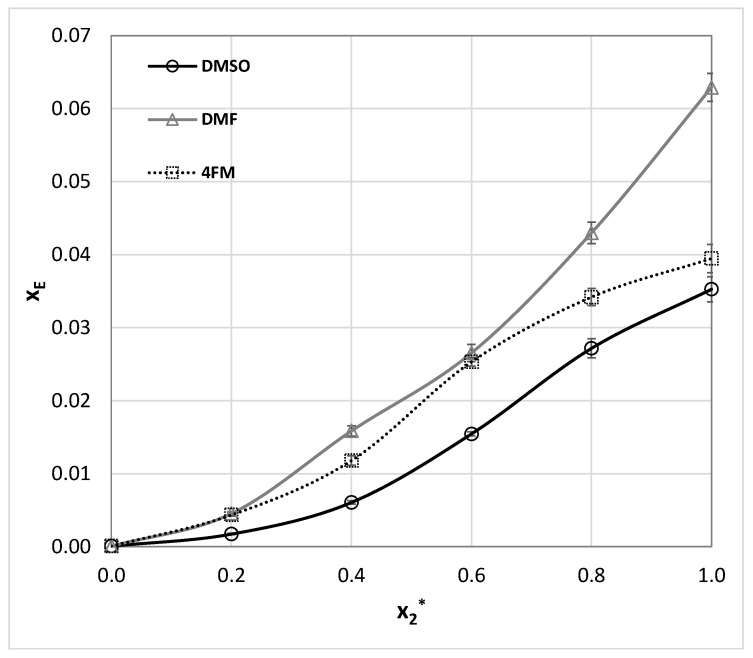
The results of ethenzamide solubility measurements (molar fraction, x_E_) at room temperature in aqueous DMSO, DMF, and 4FM mixtures (x_2_* stands for the organic component mole fraction in the binary solvent). The error bars denote standard deviation values (*n* = 3).

The solubility of this aromatic amide is available in 18 neat solvents as enumerated in Table 1. Additionally there were reported solubility values for three binary mixtures of acetonitrile with either methanol, ethanol or isopropanol [103]. Noteworthy, in all cases, the significant solubility enhancement through the co-solvation effect was observed, which shows the nontrivial solubilizing role of complex solvents containing both proton donating and accepting components. To extend the set of experimental data for ethenzamide three sets of aqueous binary mixtures were considered, by the analogy to measurements performed for other aromatic amides studied in this work. The obtained results were presented in a graphical way in Figure 7 for room temperature. The tabulated values can be found in supposing materials (Table S3). Considering published data the best solvent identified so far is DMF (x_E_ = 0.063 at 298.15 K). For consistency, ethenzamide solubility measurements in neat DMF were repeated and confronted with already published results by Wang et al. [108]. A comparison of these results with our data is provided in supporting materials (Figure S4) and it can be concluded that there is consistency between these two collections. As it is documented in Figure 7 all three aprotic solvents are very efficient solubilizers leading to the following sequence of the solubility: DMF > 4FM ≈ DMSO. Noteworthy, when considering the organic-aqueous mixtures no synergistic effect occurs by adding water, which plays a typical antisolvent role. 

**Figure 8:**
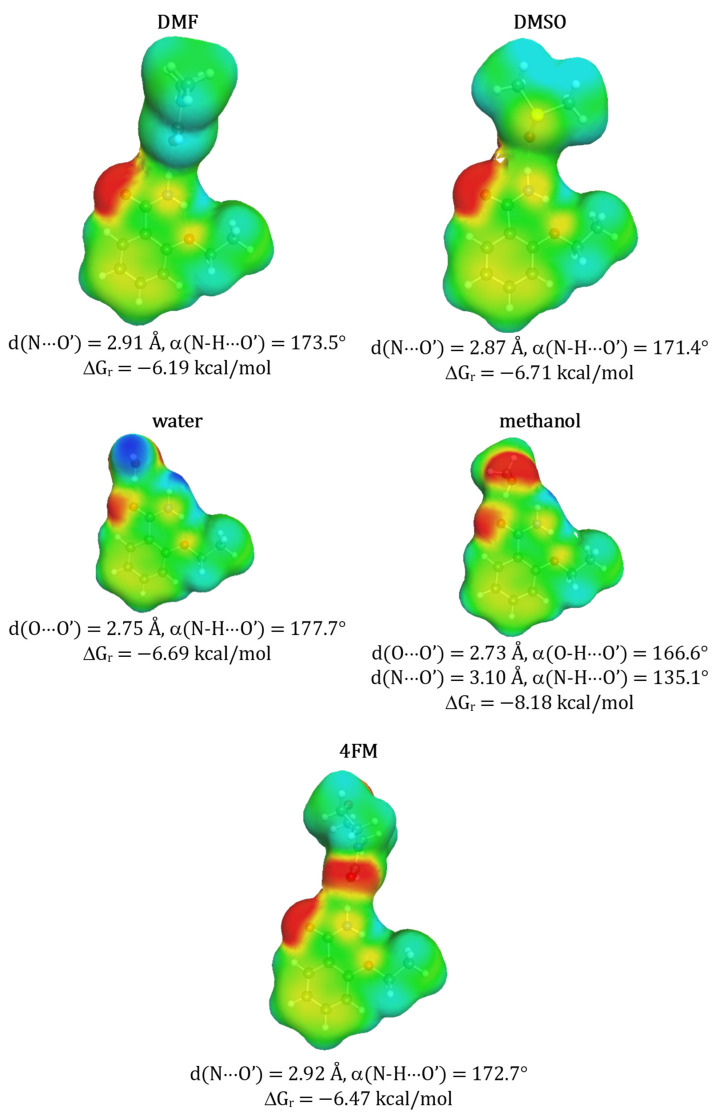
Structural and thermodynamic characteristics of selected solute solvent contacts of ethenzamide. Notation is the same as in Figure 1.

Results presented in Figure 8 suggest that ethenzamide has a lower affinity to aprotic solvents and much higher to protic ones compared to Benzamide and Salicylamide. This might be related to the steric and electrostatic hindrance imposed by the presence of an ethoxy group at orto-position and the formation of strong intramolecular hydrogen bond stabilizing ethenzamide which was already documented [10]. Consequently, the heteromolecular contacts of ethenzamide with aprotic solvent molecules have different structures than the ones observed for the other two aromatic amides. Furthermore, a very close distance between the oxygen atom of the ethoxy substituent and the amide group results in reducing the donating character of the hydrogen atom located on the latter. Consequently, all interactions with proton accepting molecules are significantly less favorable compared to benzamide and salicylamide. On the contrary, the oxygen atom of the amide group of ethenzamide acts as a stronger proton accepting center favoring hydrogen bonding with proton donating molecules, such as water and methanol. This is in accordance with the ΔGr values obtained for protic solvents. However, in general, the affinities of ethenzamide to solvents molecules are significantly lower than in case of benzamide and salicylamide which is also associated with lower solubility.

**Figure 9:**
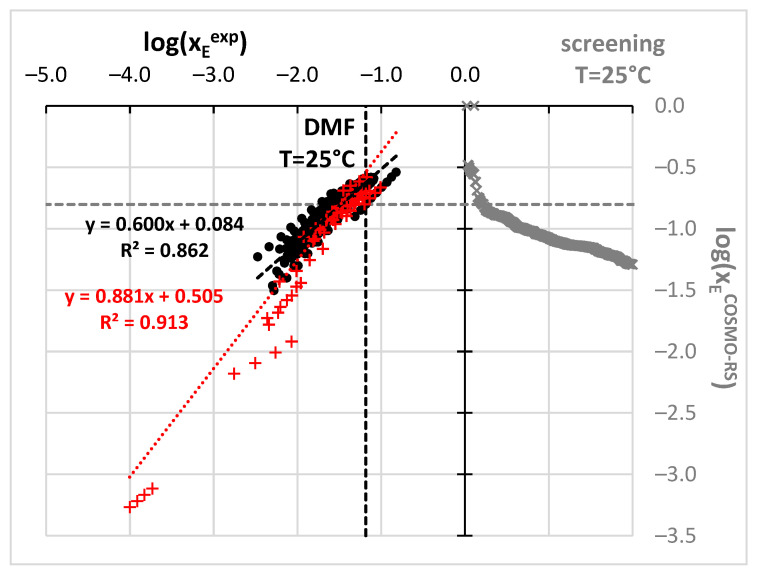
Results of ethenzamide solubility measurements and computations. Notation is the same as in Figure 2.

To complete the analysis of ethenzamide solubility the COSMO-RS modeling was performed and the obtained results are collected in Figure 9. It is evident that the quality of theoretical prediction is much lower in this case. Difficulties in the prediction of ethenzamide solubility by using COSMO-RS were already noticed [93] and a more sophisticated approach is required for achieving adequate correspondence between computed and experimental data. Despite the poorer performance of the computational protocol compared to other aromatic amides, it is still possible to use the calculation results as qualitative guidance, and all aprotic solvents used for ethenzamide solubility measurements were properly identified.

## 3. Materials and Methods

### 3.1. Chemicals

All chemicals applied in this study were purchased from commercial suppliers and used without purification. benzamide (CAS: 55-21-0, 99%), salicylamide (CAS: 65-45-2, 99%) and ethenzamide (CAS: 938-73-8, 97%) and 4-formylmorpholine (CAS: 4394-85-8, 99%) were purchased from Sigma-Aldrich (Poznań, Poland). dimethyl sulfoxide (CAS: 67-68-5, ≥99.7) and N,N-dimethylformamide (DMF, CAS: 68-12-2, ≥99.8%) were obtained from Avantor (Gliwice, Poland). Methanol (CAS: 67-56-1, ≥99.5%) was purchased from (Chempur, Piekary Slaskie, Poland). The nitrogen (99.999%) used for the DSC measurements was supplied by Linde (Warsaw, Poland).

### 3.2. Solubility Measurements Procedure

In this study, the “shake-flask” procedure used in the previous studies [18,33,40,45,47,55,61] was applied and it consists of the following steps. First, the mixtures (suspensions) containing saturated solution and undissolved solids were prepared in glass screw test tubes. Then the mixtures were incubated and agitated for 24 h at controlled temperature (298.15, 303.15, 308.15, or 313.15 K) and 60 rpm using Orbital Shaker ES-20/60 (Biosan, Riga, Latvia). At the next step, the temperature was still controlled, however, the agitation was turned off and suspensions were let to settle down for an hour. Then, the supernatant was filtrated using 0.22 μm PTFE syringe membrane filter. Nextly, 0.1 mL of the filtrate was diluted with 2 mL of methanol. The samples prepared in this way were protected against crystallization, as the concentration was low. 0.5 mL of the filtrate was used for density measurements. All equipment including automatic pipette tips, membrane filters, and syringes were preheated at the measurement temperature in order to avoid the solute precipitation. All “shake-flask” experiments were carried out in triplicate. The concentration in the samples was determined spectrophotometrically using the calibration curve method. The selected absorption maximum (λ_max_) was 223, 302 and 290 nm for benzamide, salicylamide and ethenzamide, respectively. All UV spectra were recorded using an A360 UV-VIS spectrophotometer (AOE Instruments, Shanghai, China).

### 3.3. Solid Residues Analysis

The solid residues obtained after “shake-flask” procedure were dried on air and used for IR spectrophotometric and DSC measurements. Infrared spectra were recorded using PerkinElmer (Waltham, MA, USA) spectrophotometer equipped with a diamond attenuated total reflection (ATR) device. DSC thermograms were recorded using DSC 6000 Perkin Elmer (Waltham, MA, USA) calorimeter (heating rate: 5 K/min, nitrogen flow: 20 mL/min) calibrated using indium and zinc reference standards provided by the manufacturer. 

### 3.4. Calculation Details

Theoretical characteristics of analyzed compounds started with conformational analysis for adequate representation of structural diversity. The initial structures were taken from the public PubChem database [151] and processed using the BIOVIA COSMOconf 2020 program [152] dedicated to generating the most energetically favorable conformations. The algorithm performs a series of optimizations and a number of structures reduction leading to the most probable conformers. The working part utilizes BIOVIA TURBOMOLE 2021 (release V7.5.1) [153] for geometry optimizations. The level of theory used at this stage corresponded to RI-DFT BP86 (B88-VWN-P86) with def-TZVP basis set for geometry optimization and def2-TZVPD basis set for single-point calculations with the inclusion of the fine grid tetrahedron cavity and inclusion of parameter sets with hydrogen bond interaction and van der Waals dispersion term based on the “D3” method of Grimme et al. [154]. The solubility calculations were performed using BIOVIA COSMOtherm 2021 [152] with BP_TZVPD_FINE_21.ctd parametrization. The protocol for solubility computations relies on iteratively solving the following equation,

(1) $$ln\left(\gamma_{i}^{sat,i+1}x_{i}^{sat,i+1}\right)=\frac{1}{RT}\left(\mu_{i}^{o,liq}-\mu_{i}^{\left(i\right)}\left(\gamma_{i}^{sat,i}x_{i}^{sat,i}\right)+\max\left(0,{∆}_{fus}{\bar{G}}_{i}^{m}\right)\right)$$

where superscripts *i* and *i* + 1 denote the values obtained in two subsequent iterations of solute chemical potential $\mu_{i}^{\left(i\right)}\left(a_{i}^{sat,i}\right),\;a_{i}^{sat}=\gamma_{i}^{sat}x_{i}^{sat}$ defines activity, activity coefficients, and molar fraction solubility, $\mu_{i}^{o,liq}$.stands for solute chemical potential in the liquid phase and ${∆}_{fus}{\bar{G}}_{i}^{m}$ is the partial molar Gibbs energy of melting at the solubility measurements conditions. Since COSMO-RS theory was formulated for treating bulk phases the last value is to be provided as the additional input value. There is extended discussion [47,155,156,157,158] on how properly define this quantity. Here, the simplest approach was adopted by assuming that contribution coming from the values of capacity change upon melting is small and negligible. Hence, only melting temperature and heat of fusion are necessary for estimation of the values of Gibbs free energy of fusion. These values were taken from literature as averaged values of compiled by Acree at al. [159], namely for benzamide T_m_ = 401.0 K, H_fus_ = 20.9 kcal/mol, for salicylamide T_m_ = 412.3 K, H_fus_ = 28.4 kcal/mol, and for ethenzamide T_m_ = 405.0 K, H_fus_ = 20.4 kcal/mol.

Apart from solubility, the values of solute-solvent affinities were estimated similarly as in our previous studies [33,40,55] and here only brief remarks are provided. The affinity represents the values of Gibbs free energies of hetero-association reaction X + Y = XY, where X and Y stand either for solute or solvent molecules. The extended conformational analysis precedes the actual thermodynamic computations. Many potential bimolecular clusters were considered by taking into account molecular surface segments statistics invoked in COSMOtherm program via command “CONTACT = {1 2} ssc_probability ssc_weak ssc_ang = 15.0”. These structures were further underwent geometry optimization and clustering on the same level as monomers. Finally, the values of the equilibrium constant were computed with the inclusion of energies correction accounting for zero-point vibrational energy (RI-DFT BP86 (B88-VWN-P86)/def2-TZVPD level) and electron correlation (RI-MP2/def2-QZVPP level). The solute-solvent affinity was represented by concentration-independent activity-based values of the Gibbs free energy.

## 4. Conclusions

Pharmaceutically active compounds are very often examined in terms of their solubility in various one- and multi-component solvents. In this study the COSMO-RS solubility predictions of benzamide, salicylamide, and ethenzamide were combined with green solvents selection strategy. As was established, the landscape of environmentally friendly solvents characterized by the highest solubilization abilities comprises aprotic solvents. This fact is supported by much higher stability of the interactions formed with the amide group acting as a hydrogen bond donor than when it plays an acceptor role.

Despite certain limitations of the COSMO-RS model, which was of significant importance in the case of ethenzamide, the proposed approach of selecting green solvents characterized by high solubilizing abilities seems to be reasonable. However, further research on other classes of compounds is needed. Noteworthy, 4FM, which is considered green alternative for DMF was found to be a quite efficient solvent, especially for salicylamide and ethenzamide. Moreover, in the case of the former amide, a significant co-solvation effect was observed for the aqueous binary solvent (x_s_ = 0.6), which is also expected to be more environmentally friendly than pure DMF. This is a good example of how beneficial the co-solvation effect is in terms of selecting environmentally friendly substitutes for toxic solvents. Therefore, the use of molecular modeling to select the most effective green solvents from the list including the criteria proposed by EPA, and extending the research to experimental measurements of solubility in aqueous-organic mixtures is a useful and reliable strategy.

## Figures and Tables

**Table 1:** List of neat solvents for which experimental solubility of studied aromatic amides were experimentally determined.

Neat Solvent	Benzamide	Salicylamide	Ethenzamide
water	[9]	[104]	
methanol	[9]	[104,105]	[103,106]
ethanol	[9]	[107]	[103,106]
1-propanol	[9]	[107]	[106]
isopropanol	[9]	[107]	[103,108]
1-butanol	[9]	[107]	[108]
2-butanol		[107]	[108]
isobutanol	[9]	[107]	[106]
2-methyl-2-Propanol		[107]	
1-pentanol		[107]	[108]
3-methyl-1-butanol		[107]	
1-hexanol		[107]	
1-heptanol		[107]	
1-octanol		[107]	
2-ethyl-1-hexanol		[107]	
1-decanol		[107]	
methyl acetate	[9]	[107]	[108]
ethyl acetate	[9]	[104,105]	[106]
ethyl formate			[108]
propyl acetate		[107]	[108]
butyl acetate	[9]	[107]	[108]
acetonitrile	[9]	[104,105]	[103,106]
DMF			[108]
tetrahydrofuran		[107]	
acetic acid		[104]	
acetone	[9]	[105]	[108]
1,4-dioxane		[107]	[108]
2-butanone			[108]
m-xylene	[109]		
dibutyl ether		[107]	

**Table 2:** The ranking of environmental friendliness of considered aqueous-organic solvents according to PARIS III tool [146,147,148,149,150]. The default impact factor, namely 5 was applied. The x_2_ symbol denotes the organic component molar fraction in the binary mixture. In the parentheses, the values calculated without including the PCOP parameter were provided.

Solvent	X_2_	HTPIng × 10	HTPInh	TTP	ATP × 10^5^	GWP	ODP	PCOP	AR	EI	Rank
DMSO	0.2	0.73	0.00	0.07	3.23	0.0	0.0	5.94	0.0	6.09 (0.15)	12(2)
	0.4	0.99	0.00	0.10	4.61	0.0	0.0	8.49	0.0	8.69 (0.20)	13(3)
	0.6	1.14	0.00	0.11	5.38	0.0	0.0	9.90	0.0	10.10 (0.23)	14(4)
	0.8	1.24	0.00	0.12	5.87	0.0	0.0	10.80	0.0	11.00 (0.25)	15(5)
	1.0	1.30	0.00	0.13	6.20	0.0	0.0	11.40	0.0	11.70 (0.26)	16(6)
DMF	0.2	3.45	0.41	0.35	10.10	0.0	0.0	0.00	0.0	1.10 (1.10)	7(12)
	0.4	4.96	0.59	0.50	14.60	0.0	0.0	0.00	0.0	1.58 (1.58)	8(13)
	0.6	5.81	0.69	0.58	17.20	0.0	0.0	0.00	0.0	1.85 (1.85)	9(14)
	0.8	6.36	0.76	0.64	18.90	0.0	0.0	0.00	0.0	2.03 (2.03)	10(15)
	1.0	6.75	0.81	0.68	20.00	0.0	0.0	0.00	0.0	2.16 (2.16)	11(16)
4FM	0.2	1.60	0.00	0.16	61.40	0.0	0.0	0.00	0.0	0.32 (0.32)	2(7)
	0.4	2.07	0.00	0.21	80.90	0.0	0.0	0.00	0.0	0.42 (0.41)	3(8)
	0.6	2.31	0.00	0.23	90.50	0.0	0.0	0.00	0.0	0.46 (0.46)	4(9)
	0.8	2.44	0.00	0.24	96.10	0.0	0.0	0.00	0.0	0.49 (0.49)	5(10)
	1.0	2.54	0.00	0.25	99.90	0.0	0.0	0.00	0.0	0.51 (0.51)	6(11)

## Data Availability

All available data were provided in the manuscript and Supplementary Materials.

## Supplementary Materials

The following supporting information can be downloaded at: https://www.mdpi.com/article/10.3390/molecules27103323/s1, Figure S1: The comparison of benzamide solubility profiles in binary solvents containing DMSO (a), DMF (b), 4FM (c). The organic component mole fraction in the binary solvent was denoted by x_2_*; Table S1: Values of benzamide molar fraction solubility (x_B_) in binary aqueous-organic solvents. x_2_* stands for the organic component mole fraction in the binary solvent; Figure S2: The comparison of salicylamide solubility profiles in binary solvents containing DMSO (a), DMF (b), 4FM (c). The organic component mole fraction in the binary solvent was denoted by x_2_*; Table S2: Values of salicylamide molar fraction solubility (x_S_) in binary aqueous-organic solvents. x_2_* stands for the organic component mole fraction in the binary solvent; Figure S3: The comparison of ethenzamide solubility profiles in binary solvents containing DMSO (a), DMF (b), 4FM (c). The organic component mole fraction in the binary solvent was denoted by x_2_*; Table S3: Values of ethenzamide molar fraction solubility (x_E_) in binary aqueous-organic solvents. x_2_* stands for the organic component mole fraction in the binary solvent; Figure S4: The comparison of ethenzamide solubility data in DMF obtained in this study and reported in the literature (Wang et al., J. Chem. Eng. Data 2021, 66, 1508–1514); Figure S5: The FTIR-ATR spectra recorded for the benzamide sediments collected after shake-flask experiments; Figure S6: The FTIR-ATR spectra recorded for the salicylamide sediments collected after shake-flask experiments; Figure S7: The FTIR-ATR spectra recorded for the ethenzamide sediments collected after shake-flask experiments; Figure S8: The DSC thermograms recorded for the benzamide sediments collected after shake-flask experiments; Figure S9: The DSC thermograms recorded for the salicylamide sediments collected after shake-flask experiments; Figure S10: The DSC thermograms recorded for the ethenzamide sediments collected after shake-flask experiments.
