# Supplementary material for: New Screening Protocol for Effective Green Solvents Selection of Benzamide, Salicylamide and Ethenzamide
Source: Molecules. 2022 May 22;27(10):3323. doi: 10.3390/molecules27103323 (PMC9144492; doi:10.3390/molecules27103323)
Supplement: Supplementary file 1 [file molecules-27-03323-s001.zip › Supplementary Materials.pdf]

Supplementary Materials

New screening protocol for effective green solvents selection of benzamide, salicylamide and ethenzamide

Maciej Przybyłek, Anna Miernicka, Mateusz Nowak and Piotr Cysewski**
Department of Physical Chemistry, Pharmacy Faculty, Collegium Medicum of Bydgoszcz, Nicolaus Copernicus University in Toruń, Kurpińskiego 5, 85-950 Bydgoszcz, Poland; m.przybylek@cm.umk.pl (M.P.); 282892@stud.umk.pl (A.M); 294597@stud.umk.pl (M.N.); Piotr.Cysewski@cm.umk.pl (P.C.)
**Correspondence: m.przybylek@cm.umk.pl (M.P.); Piotr.Cysewski@cm.umk.pl (P.C.)*

Table of contents

SI. The benzamide, salicylamide and ethenzamide solubility values determined for DMSO, DMF and 4FM and their mixtures with water 3

Figure S1. The comparison of benzamide solubility profiles in binary solvents containing DMSO (a), DMF (b), 4FM (c). The organic component mole fraction in the binary solvent was denoted by x_2^*. 3

Table. S1. Values of benzamide molar fraction solubility (x_B) in binary aqueous-organic solvents. x_2^* stands for the organic component mole fraction in the binary solvent. 4

Figure S2. The comparison of salicylamide solubility profiles in binary solvents containing DMSO (a), DMF (b), 4FM (c). The organic component mole fraction in the binary solvent was denoted by x_2*. 5

Table. S2. Values of salicylamide molar fraction solubility (x_S) in binary aqueous-organic solvents. x_2^* stands for the organic component mole fraction in the binary solvent. 6

Figure S3. The comparison of ethenzamide solubility profiles in binary solvents containing DMSO (a), DMF (b), 4FM (c). The organic component mole fraction in the binary solvent was denoted by x_2^*. 7

Table. S3. Values of ethenzamide molar fraction solubility (x_E) in binary aqueous-organic solvents. x_2^* stands for the organic component mole fraction in the binary solvent. 8

Figure S4. The comparison of ethenzamide solubility data in DMF obtained in this study and reported in the literature (Wang et al., J. Chem. Eng. Data 2021, 66, 1508–1514). 9

SII. The FTIR-ATR characteristics of sediments 10

Figure S5. The FTIR-ATR spectra recorded for the benzamide sediments collected after shake-flask experiments 10

Figure S6. The FTIR-ATR spectra recorded for the salicylamide sediments collected after shake-flask experiments 11

Figure S7. The FTIR-ATR spectra recorded for the ethenzamide sediments collected after shake-flask experiments 12

SIII. The DSC characteristics of sediments 13

Figure S8. The DSC thermograms recorded for the benzamide sediments collected after shake-flask experiments.. 13

Figure S9. The DSC thermograms recorded for the salicylamide sediments collected after shake-flask experiments.. 14

Figure S10. The DSC thermograms recorded for the ethenzamide sediments collected after shake-flask experiments.. 15

SI. The benzamide, salicylamide and ethenzamide solubility values determined for DMSO, DMF and 4FM and their mixtures with water

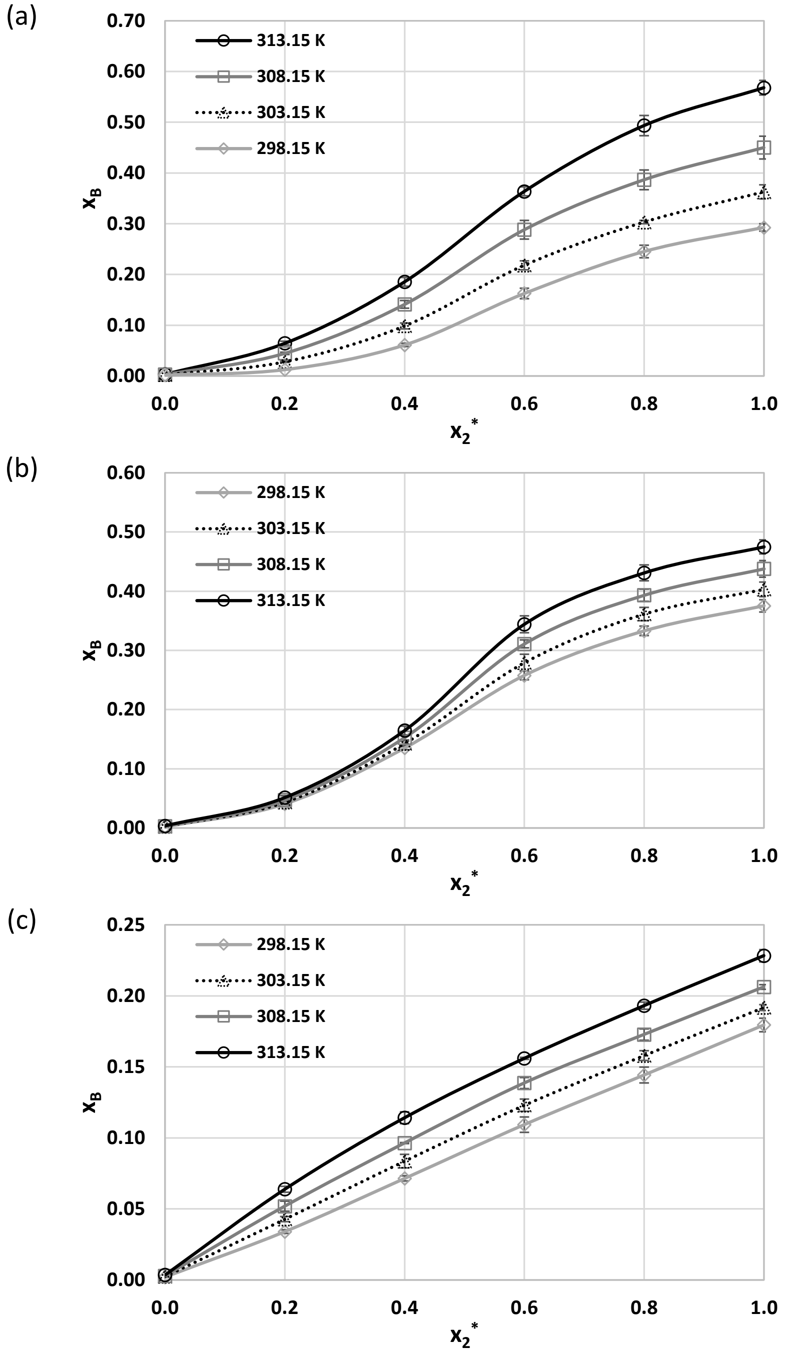

Figure S1. The comparison of benzamide solubility profiles in binary solvents containing DMSO (a), DMF (b), 4FM (c). The organic component mole fraction in the binary solvent was denoted by x_2^*.

Table. S1. Values of benzamide molar fraction solubility (x_B) in binary aqueous-organic solvents. x_2^* stands for the organic component mole fraction in the binary solvent.

T [K]	x_2^*	$x_B \times 10^3$		
		**DMSO**	**DMF**	**4FM**
298.15	0.0	2.03±0.03	2.03±0.03	2.03±0.03
	0.2	12.76±0.69	40.84±1.34	34.03±1.20
	0.4	61.24±3.30	135.13±2.92	71.44±1.78
	0.6	162.69±10.34	257.80±7.53	109.36±5.43
	0.8	245.28±12.34	332.95±8.10	144.27±5.63
	1.0	292.73±7.24	375.39±10.37	179.49±4.76
303.15	0.0	2.50±0.16	2.50±0.16	2.50±0.16
	0.2	28.12±1.48	43.48±1.18	42.81±1.82
	0.4	98.76±5.79	142.71±3.86	83.65±4.84
	0.6	218.23±8.94	279.22±14.27	122.99±4.29
	0.8	303.32±3.25	361.33±11.14	157.94±3.50
	1.0	362.74±13.83	403.22±12.29	191.76±2.05
308.15	0.0	2.94±0.09	2.94±0.09	2.94±0.09
	0.2	44.76±2.39	47.21±0.92	51.96±3.49
	0.4	141.17±7.63	152.71±3.96	96.42±0.35
	0.6	288.35±18.43	310.97±6.76	138.65±3.87
	0.8	386.75±19.39	393.13±10.37	172.78±3.98
	1.0	450.15±22.39	437.73±14.13	206.23±1.67
313.15	0.0	3.53±0.09	3.53±0.09	3.53±0.09
	0.2	64.64±3.79	51.30±3.30	63.89±2.12
	0.4	185.70±8.48	164.58±7.29	114.17±4.21
	0.6	363.83±8.70	344.14±14.37	155.97±0.67
	0.8	493.50±19.89	430.80±13.36	193.06±2.55
	1.0	567.98±14.37	474.77±11.86	228.22±4.32

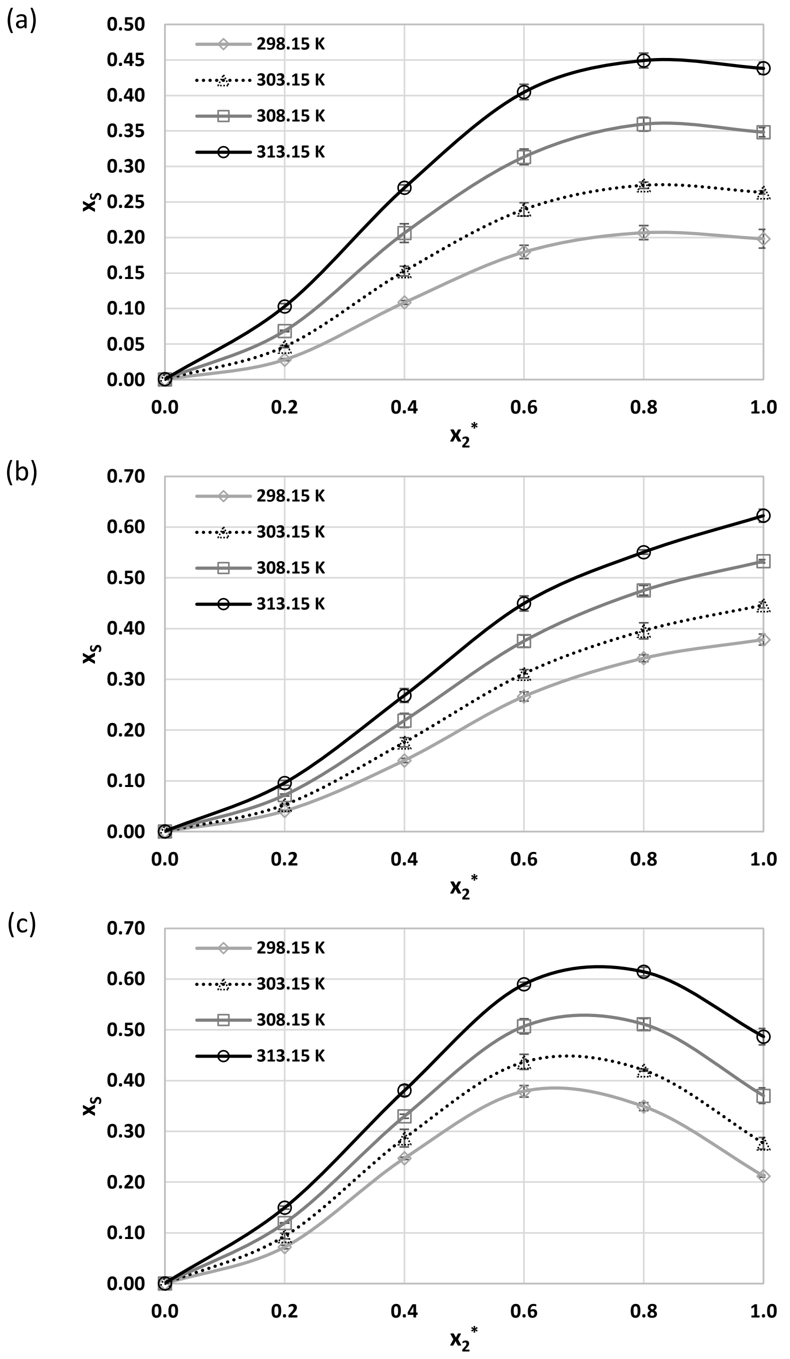

Figure S2. The comparison of salicylamide solubility profiles in binary solvents containing DMSO (a), DMF (b), 4FM (c). The organic component mole fraction in the binary solvent was denoted by x_2^*.

Table. S2. Values of salicylamide molar fraction solubility (x_S) in binary aqueous-organic solvents. x_2^* stands for the organic component mole fraction in the binary solvent.

		$x_S \times 10^3$		
**T [K]**	x_2^*	**DMSO**	**DMF**	**4FM**
298.15	0.0	0.31±0.01	0.31±0.01	0.31±0.01
	0.2	28.16±1.50	40.70±1.51	72.09±3.37
	0.4	108.62±2.77	140.29±4.17	247.04±2.29
	0.6	179.70±9.24	266.44±9.12	379.01±11.19
	0.8	206.85±9.95	341.69±6.79	348.86±7.70
	1.0	198.28±13.18	378.44±10.91	211.91±2.02
303.15	0.0	0.39±0.01	0.39±0.01	0.39±0.01
	0.2	46.44±1.38	53.11±0.71	93.66±5.63
	0.4	152.68±6.95	176.10±9.00	286.65±17.42
	0.6	239.62±9.27	311.34±8.09	436.90±14.76
	0.8	273.59±4.34	395.85±15.89	420.73±2.32
	1.0	263.53±2.39	446.40±5.87	276.59±11.24
308.15	0.0	0.50±0.03	0.50±0.03	0.50±0.03
	0.2	68.57±1.09	72.19±1.90	119.62±0.39
	0.4	206.30±13.23	219.09±13.72	329.92±4.10
	0.6	313.43±11.01	375.58±11.86	507.09±14.88
	0.8	359.50±9.68	475.35±9.61	511.12±11.54
	1.0	348.26±6.46	532.79±3.25	370.33±15.39
313.15	0.0	0.62±0.02	0.62±0.02	0.62±0.02
	0.2	103.16±3.80	96.03±4.81	149.67±2.95
	0.4	270.05±4.06	268.21±13.64	380.58±10.34
	0.6	404.76±10.80	449.64±14.83	590.16±4.01
	0.8	449.30±10.48	550.81±4.42	614.56±8.51
	1.0	438.19±8.64	622.64±12.83	486.78±16.14

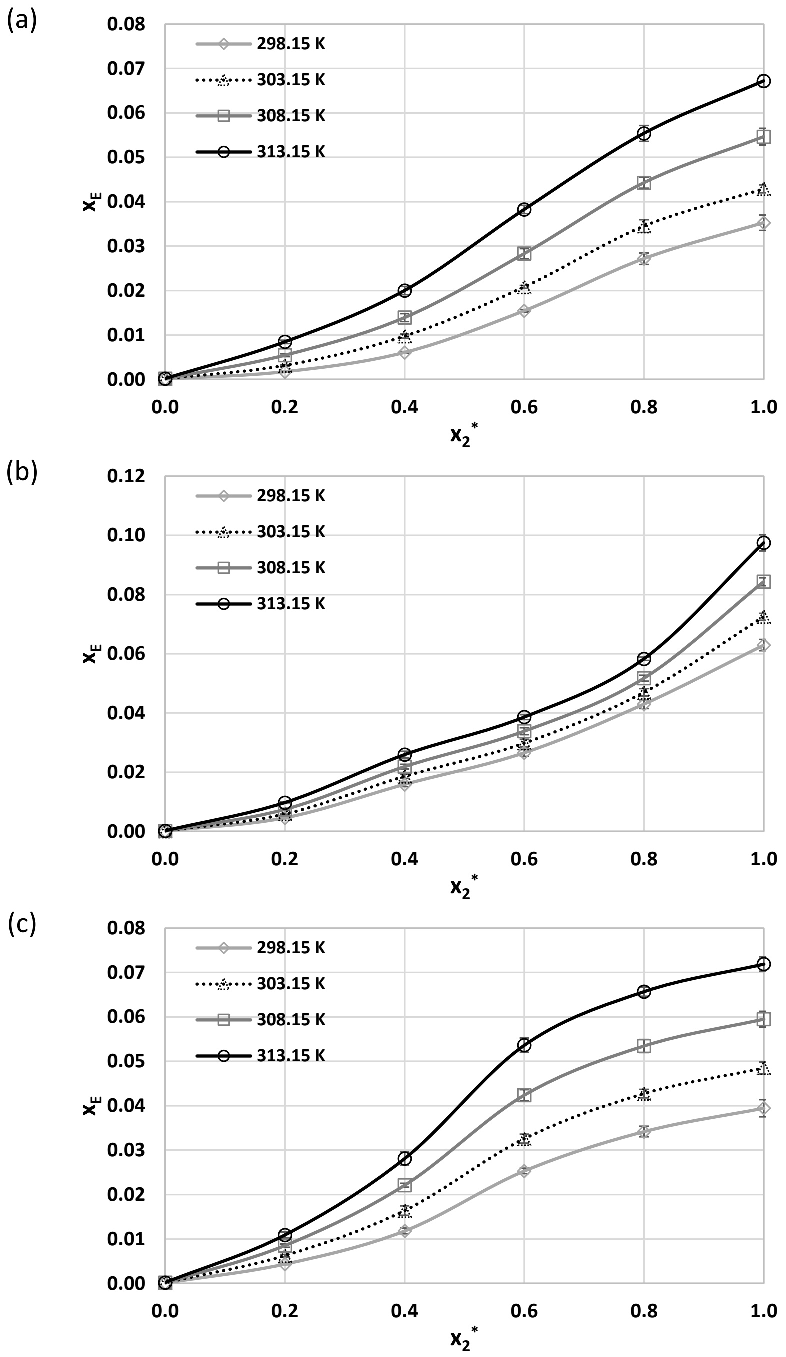

Figure S3. The comparison of ethenzamide solubility profiles in binary solvents containing DMSO (a), DMF (b), 4FM (c). The organic component mole fraction in the binary solvent was denoted by x_2^*.

Table. S3. Values of ethenzamide molar fraction solubility (x_E) in binary aqueous-organic solvents. x_2^* stands for the organic component mole fraction in the binary solvent.

		$x_E \times 10^3$		
**T [K]**	**x_2^***	**DMSO**	**DMF**	**4FM**
298.15	0.0	0.10±0.00	0.10±0.00	0.10±0.00
	0.2	1.74±0.08	4.54±0.20	4.35±0.15
	0.4	6.08±0.22	15.86±0.72	11.81±0.64
	0.6	15.46±0.28	26.54±1.18	25.33±0.58
	0.8	27.18±1.31	42.98±1.46	34.19±1.18
	1.0	35.27±1.72	62.90±1.91	39.47±1.93
303.15	0.0	0.12±0.00	0.12±0.00	0.12±0.00
	0.2	3.13±0.13	5.86±0.11	6.24±0.29
	0.4	9.72±0.46	18.59±0.67	16.40±1.05
	0.6	20.83±0.30	29.82±1.27	32.59±0.99
	0.8	34.52±1.44	46.96±1.26	42.75±0.94
	1.0	42.93±0.90	72.50±1.19	48.50±1.36
308.15	0.0	0.15±0.01	0.15±0.01	0.15±0.01
	0.2	5.45±0.30	7.45±0.31	8.52±0.37
	0.4	13.92±0.91	21.89±0.80	22.11±0.45
	0.6	28.33±1.13	33.87±1.16	42.40±1.23
	0.8	44.29±1.24	51.74±1.00	53.47±1.47
	1.0	54.65±1.87	84.33±1.36	59.51±1.75
313.15	0.0	0.19±0.01	0.19±0.01	0.19±0.01
	0.2	8.45±0.36	9.71±0.26	10.90±0.68
	0.4	20.01±0.93	25.91±1.11	28.10±1.52
	0.6	38.27±0.90	38.61±0.85	53.63±1.61
	0.8	55.40±1.79	58.30±0.54	65.71±1.06
	1.0	67.21±1.28	97.49±2.70	71.91±1.62

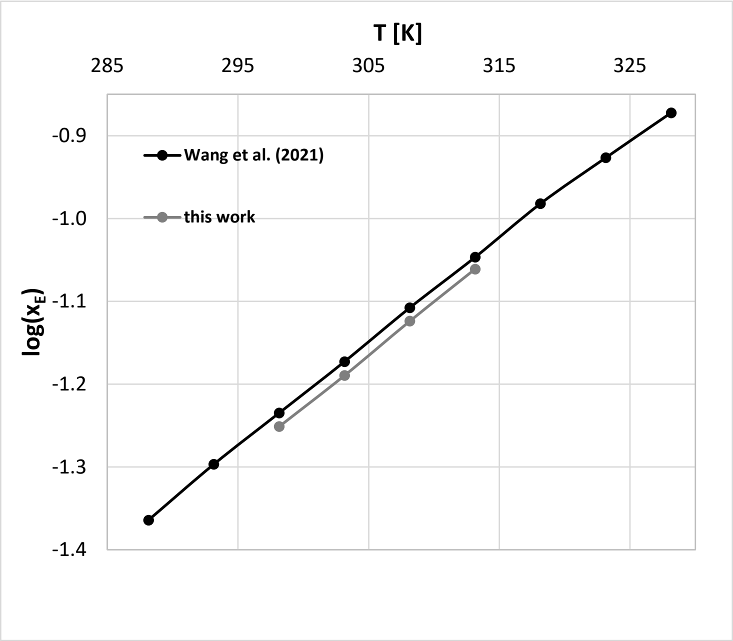

Figure S4. The comparison of ethenzamide solubility data in DMF obtained in this study and reported in the literature (Wang et al., J. Chem. Eng. Data 2021, 66, 1508–1514).

SII. The FTIR-ATR characteristics of sediments

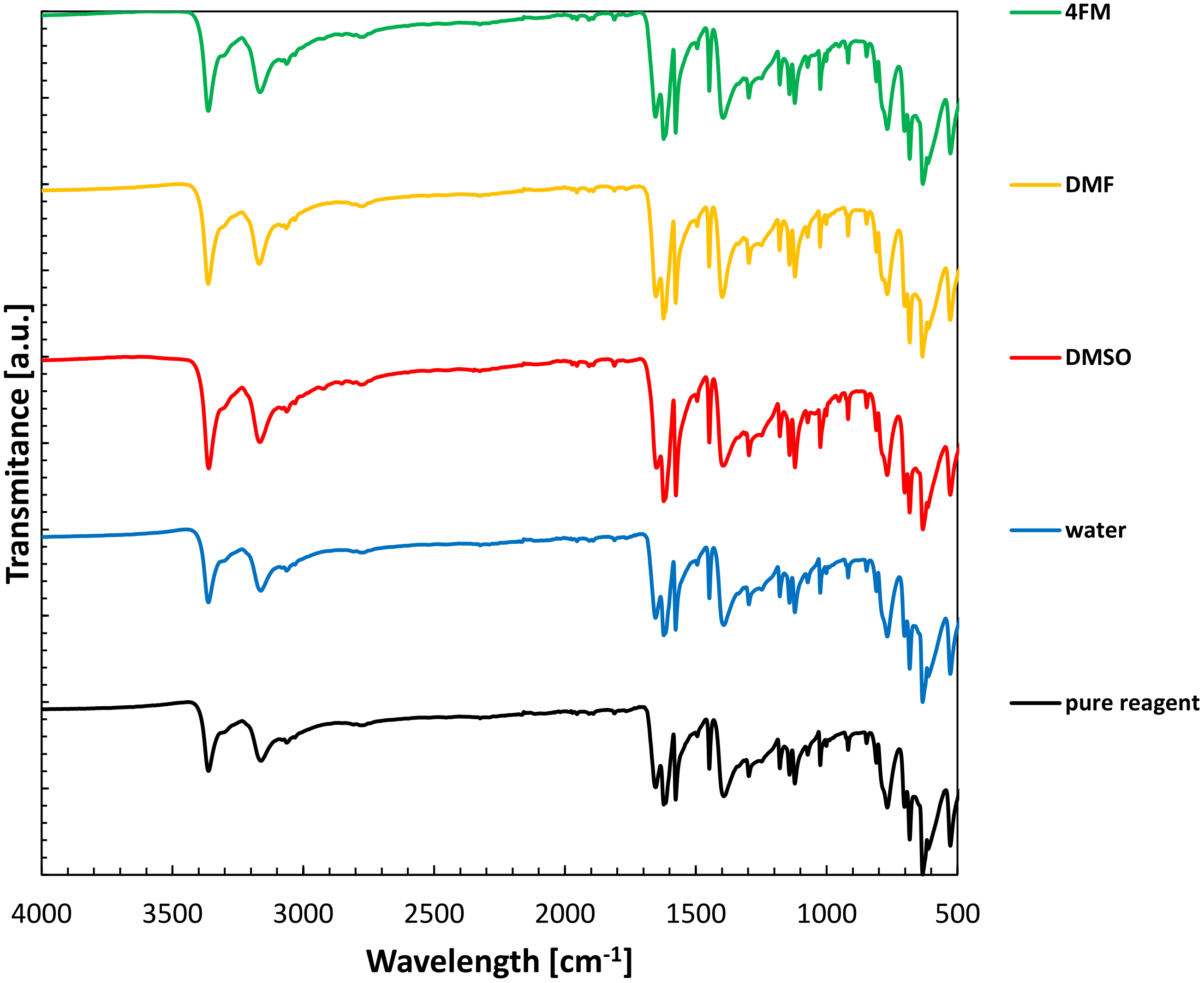

Figure S5. The FTIR-ATR spectra recorded for the benzamide sediments collected after shake-flask experiments.

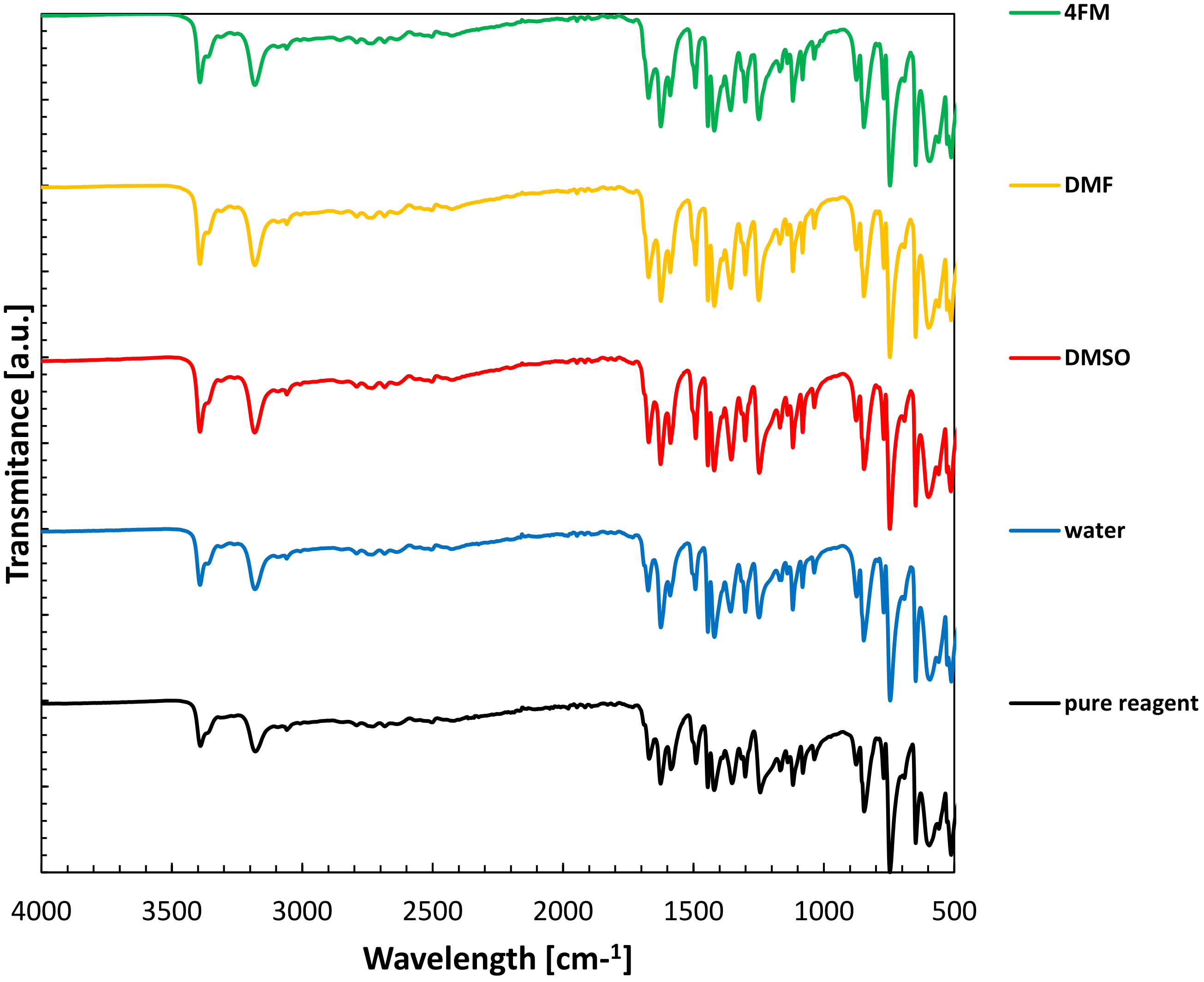

Figure S6. The FTIR-ATR spectra recorded for the salicylamide sediments collected after shake-flask experiments.

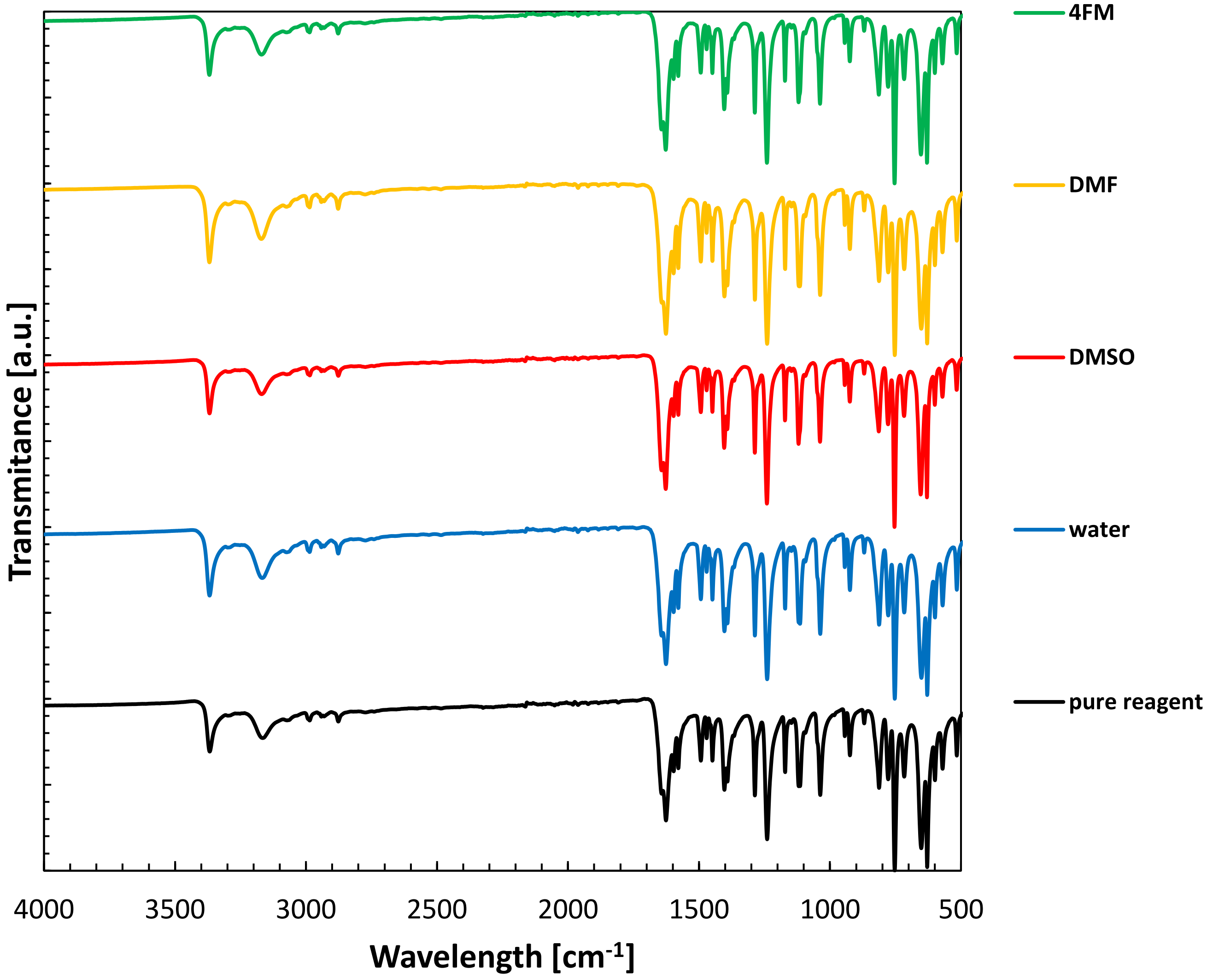

Figure S7. The FTIR-ATR spectra recorded for the ethenzamide sediments collected after shake-flask experiments.

SIII. The DSC characteristics of sediments

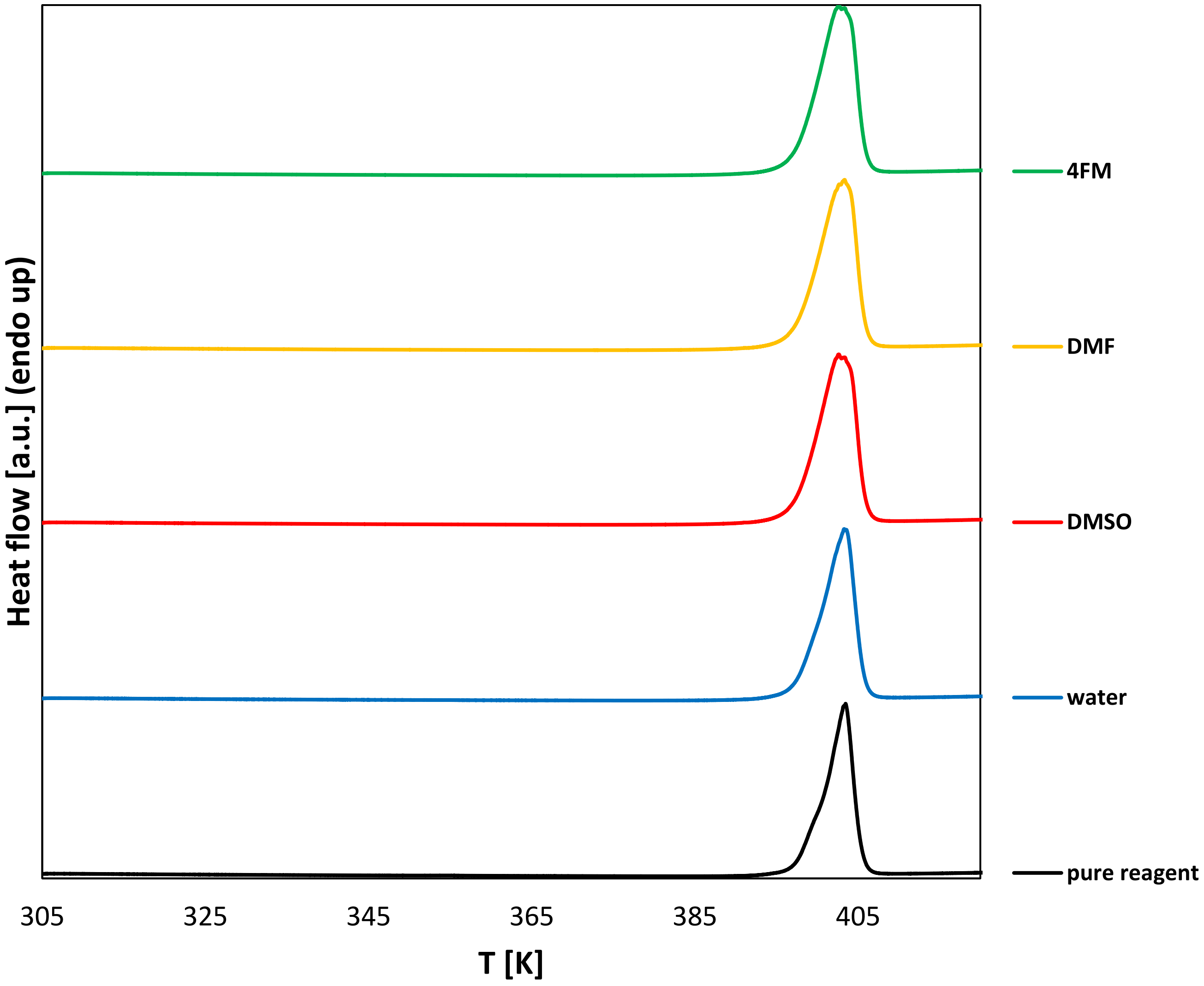

Figure S8. The DSC thermograms recorded for the benzamide sediments collected after shake-flask experiments.

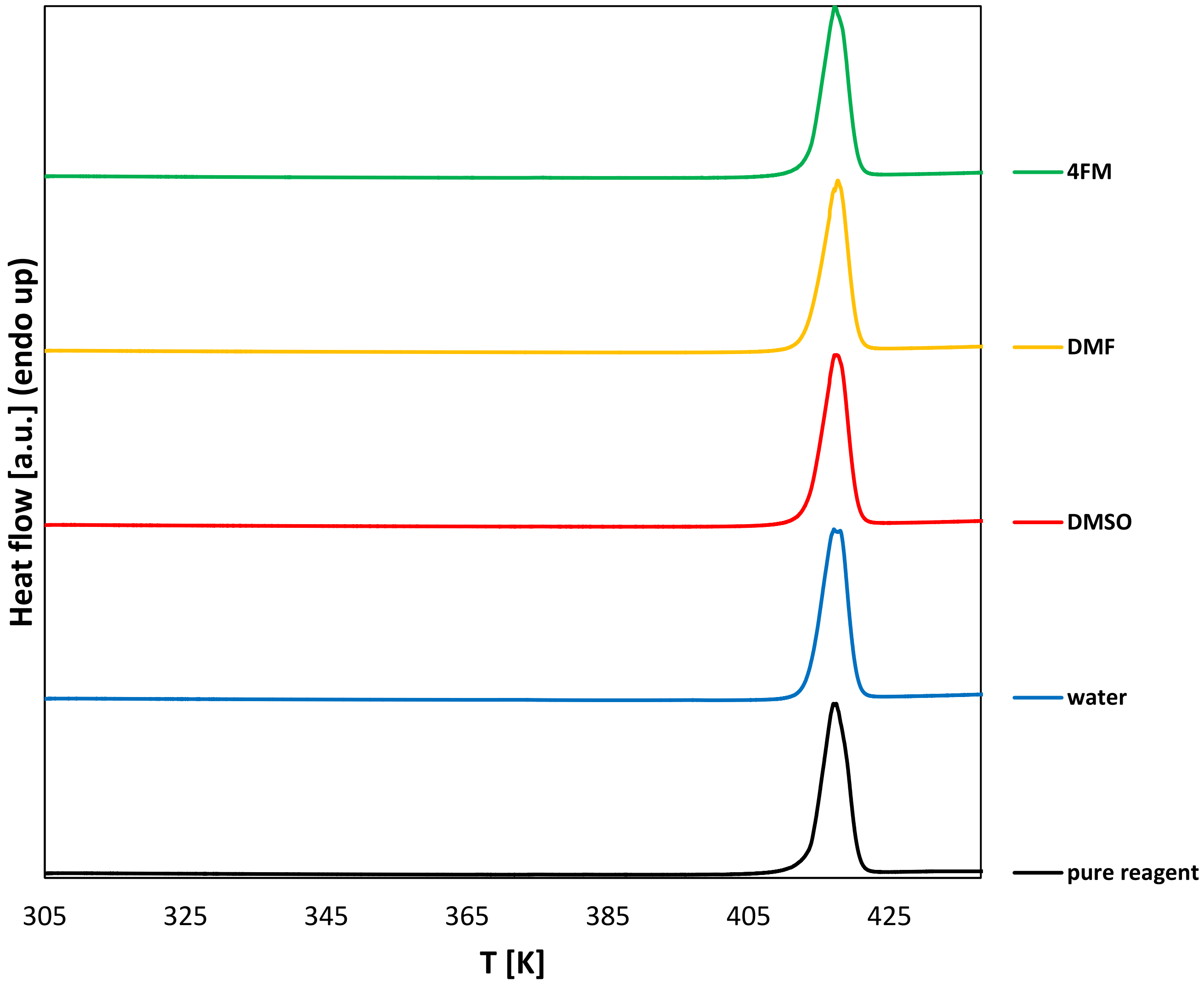

Figure S9. The DSC thermograms recorded for the salicylamide sediments collected after shake-flask experiments.

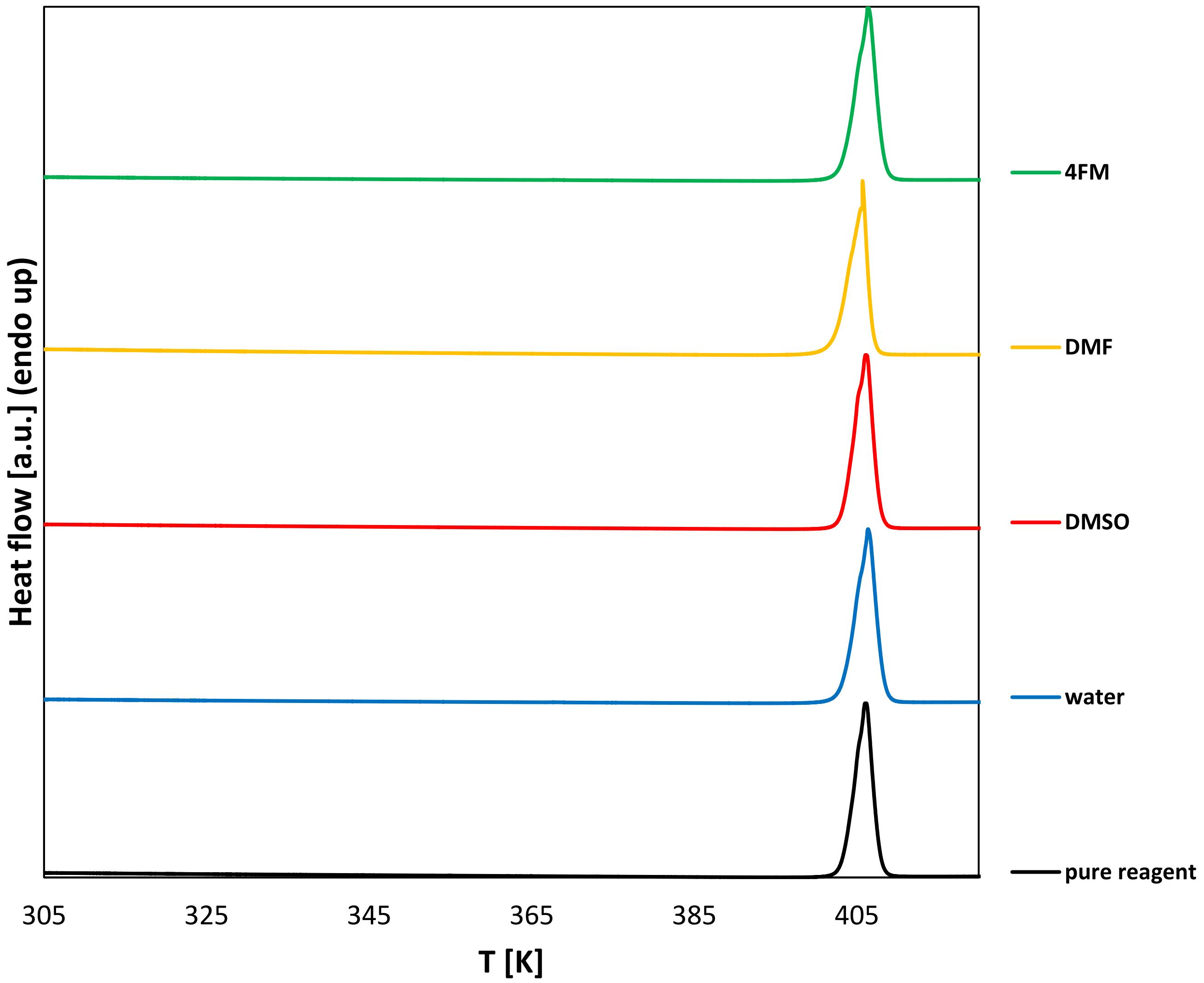

Figure S10. The DSC thermograms recorded for the ethenzamide sediments collected after shake-flask experiments.
